# Influenza Outbreaks during World Youth Day 2008 Mass Gathering

**DOI:** 10.3201/eid1605.091136

**Published:** 2010-05

**Authors:** Christopher C. Blyth, Hong Foo, Sebastiaan J. van Hal, Aeron C. Hurt, Ian G. Barr, Kenneth McPhie, Paul K. Armstrong, William D. Rawlinson, Vicky Sheppeard, Stephen Conaty, Michael Staff, Dominic E. Dwyer, on behalf of the World Youth Day 2008 Influenza Study Group

**Affiliations:** Westmead Hospital, Sydney, New South Wales, Australia (C.C. Blyth, H. Foo, S.J. van Hal, K. McPhie, D.E. Dwyer); WHO Collaborating Centre for Influenza Reference and Research, Melbourne, Victoria, Australia (A.C. Hurt, I.G. Barr); New South Wales Department of Health, Sydney (P.K. Armstrong); Prince of Wales Hospital, Sydney (W.D. Rawlinson); Sydney West Public Health Unit, Paramatta, New South Wales, Australia (V. Sheppeard); Sydney South West Public Health Unit, Camperdown, New South Wales, Australia (S. Conaty); North Sydney Central Coast Public Health Unit, Hornsby, New South Wales, Australia (M. Staff); 1 Current affiliation: University of Western Australia, Perth, Australia.; 2 Other members of the World Youth Day 2008 Influenza Study Group are listed at the end of this article.

**Keywords:** influenza, mass gatherings, outbreak, research

## Abstract

Influenza outbreaks during mass gatherings have been rarely described, and detailed virologic assessment is lacking. An influenza outbreak occurred during World Youth Day in Sydney, Australia, July 2008 (WYD2008). We assessed epidemiologic data and respiratory samples collected from attendees who sought treatment for influenza-like illness at emergency clinics in Sydney during this outbreak. Isolated influenza viruses were compared with seasonal influenza viruses from the 2008 influenza season. From 100 infected attendees, numerous strains were identified: oseltamivir-resistant influenza A (H1N1) viruses, oseltamivir-sensitive influenza A (H1N1) viruses, influenza A (H3N2) viruses, and strains from both influenza B lineages (B/Florida/4/2006-like and B/Malaysia/2506/2004-like). Novel viruses were introduced, and pre-WYD2008 seasonal viruses were amplified. Viruses isolated at mass gatherings can have substantial, complex, and unpredictable effects on community influenza activity. Greater flexibility by public health authorities and hospitals is required to appropriately manage and contain these outbreaks.

## Abstract 2

Novel viruses were introduced and seasonal viruses were amplified.

Influenza is caused by a highly infectious respiratory virus with the potential to rapidly spread in susceptible hosts. Influenza outbreaks have frequently been described in populations such as residents of nursing care facilities (*1*,*2*), residential schools or colleges (*3*,*4*), prisons (*5*), military facilities (*6*), and other enclosed communities (*7*).

Mass gatherings pose complex and unique challenges to public health and medical services. Because populations are increasingly mobile, and more able to attend large gatherings, the risk for outbreaks of influenza and other infectious diseases among a susceptible population has increased, and a substantial responsibility is placed on health services if outbreaks occur. Despite this situation, influenza outbreaks during mass gatherings have rarely been described (*8*–*10*), and reports have not included results of detailed virologic testing. Furthermore, the effects of outbreak strains on local influenza epidemiology have not been assessed.

During World Youth Day (WYD2008) celebrations, 223,000 predominately young pilgrims from 170 countries attended a series of mass religious gatherings from July 15 to July 20, 2008, in Sydney, New South Wales (NSW), Australia. At the end of the week, an outdoor evening vigil with >200,000 participants preceded the final mass presided over by Pope Benedict XVI. This mass was attended by an estimated 400,000 persons (*11*). Approximately 100,000 pilgrims were given accommodation in sporting facilities, schools, and community centers, where temporary floor mats and blankets were provided and other facilities were shared. The largest site of accommodation, the Sydney Olympic Park site, provided lodging for as many as 12,000 pilgrims each night. Pilgrims remained housed overnight at the allocated accommodation sites and attended numerous outdoor religious gatherings during the day with other pilgrims. Many pilgrims traveled in Australia and New Zealand before and after the WYD2008 celebrations, visiting major cities and rural areas.

We describe the epidemiologic and virologic features of an influenza outbreak predominantly among young adults during WYD2008 celebrations. These data provide insight into the complexity of influenza outbreaks during mass gatherings and their effects on the community at large. The insights gained should guide plans for mass events, particularly when held during periods of peak influenza activity.

## Methods

Influenza was first identified among WYD2008 pilgrims on July 16, 2008. Emergency clinics were then quickly established to identify and isolate infected pilgrims. Symptomatic pilgrims were encouraged to visit the clinics, which were open 24 hours a day. Epidemiologic data were collected prospectively from all pilgrims who sought treatment. Respiratory tract samples (paired nose and throat swabs specimens) were obtained as previously described (*12*).

Influenza testing included the following: 1) point-of-care tests (POCTs) performed either on site or in the laboratory (Quickvue A & B; Quidel, San Diego, CA, USA, or BinaxNOW Influenza A & B, Binax, Scarborough, ME, USA), 2) antigen detection using type-specific indirect fluorescent antibodies (IFA) (Chemicon, Millipore, Billerica, MA, USA, or Bartels, Immunodiagnostic Supplies Inc., Bellevue, WA, USA); 3) validated in-house type- and subtype-specific nucleic acid testing (NAT) by using PCR that targeted the matrix, nonstructural, and hemagglutinin region of the influenza virus genome; and 4) virus culture using MDCK cells (*13*). IFA and NAT were the preferred diagnostic methods. The decision to perform POCT was made on a case-by-case basis by clinicians. Viral culture was performed on antigen- (POCT/IFA) or NAT-positive specimens. Testing was performed at 2 virology laboratories (Institute of Clinical Pathology and Medical Research, Westmead Hospital, Sydney, New South Wales, Australia; South Eastern Area Laboratory Service, Prince of Wales Hospital, Randwick, New South Wales).

Pilgrims with clinical or laboratory-confirmed influenza who sought treatment within 48 hours of symptom onset were offered oseltamivir (75 mg 2×/d for 5 days). Public health authorities recommended that infected pilgrims remain in isolation for 48 hours or until 5 days after symptom onset.

All influenza isolates from WYD2008 were sent to the WHO Collaborating Centre for Reference and Research on Influenza in Melbourne, Victoria, Australia, where antigenic analysis was performed by using a hemagglutination-inhibition assay (*14*). Oseltamivir susceptibility was tested by using a fluorescence-based neuraminidase (NA) inhibition assay (*15*). When no isolate was available, clinical samples were directly tested by rolling circle amplification for the H274Y mutation (the most frequently reported mutation conferring oseltamivir resistance) (*16*). Hemagglutinin (HA) and NA gene sequencing was performed by using standard methods (*17*). Sequence alignment was performed by using ClustalW (www.ebi.ac.uk/Tools/clustalw2/index.html) in DNAstar Lasergene version 8 (www.dnastar.com), and phylogenetic trees were generated by using maximum-likelihood (DNAML) in PHYLIP (*18*).

Australia-wide laboratory-confirmed influenza data were obtained from the National Notifiable Diseases Surveillance System (*19*). The influenza viruses isolated during WYD2008 were compared with viruses submitted to the WHO Collaborating Centre from all Australian states and territories during the 2008 influenza season. HA and NA sequences from WYD2008 viruses were compared with a representative sample of seasonal influenza viruses from around the world, including Australia, sequenced by the WHO Collaborating Centre.

## Results

Respiratory tract samples were obtained from 227 WYD2008 attendees who sought treatment at established clinics. The true extent of infection is unknown because the pilgrims voluntarily visited the clinics, and respiratory tract sampling was limited at several accommodation sites after the outbreak was identified. The median age of the pilgrims tested was 21 years (range 12–72 years, interquartile range 18–28 years); 62.8% were female. Twenty-nine percent of pilgrims tested lived in Australia, and the remainder were from overseas (Europe, 28.0%; Oceania, 20.2%; North America, 17.1%; South or Central America, 2.6%; Asia, 2.6%; Africa, 0.5%). Recent influenza vaccination was infrequent; 25 (21.6%) of 116 reported recent vaccination (Southern Hemisphere, 12.0%; Northern Hemisphere, 27.9%; p = 0.021). Demographic characteristics of pilgrims who visited established clinics were not significantly different from those of the total pilgrim population (*11*).

Two or more influenza diagnostic tests were performed on all specimens (POCT, 80%; IFA, 100%; NAT, 97%; and virus culture, 43%). Laboratory confirmation of influenza virus infection was obtained for 100 (44.1%) pilgrims. This included 69 patients whose test results were positive by both antigen detection (POCT or IFA) and NAT, 21 patients with positive results by NAT yet negative by antigen detection, 5 patients with positive results by both antigen detection and viral culture, and 5 patients with positive results by 2 antigen detection methods (when clinical material was not sufficient for NAT or culture). Pilgrims had symptoms for a median of 2 days before they visited a clinic (95% confidence interval 1.7–2.7 days). No significant differences in age, sex, or country of origin were noted between the pilgrims with laboratory-confirmed cases, pilgrims whose test results were negative, and pilgrims who were not tested (data not shown).

Influenza types A and B were identified during WYD2008 (Table). Influenza A was most frequently isolated from patients from Australia and Germany, whereas influenza B was most frequently isolated from patients from the Solomon Islands, Papua New Guinea, Australia, and North America. Numerous distinct circulating influenza viruses were identified: oseltamivir-resistant influenza A (H1N1) A/Brisbane/59/2007-like viruses, oseltamivir-sensitive influenza A (H1N1) viruses (no isolate was recovered for serotyping), influenza A (H3N2) A/Brisbane/10/2007-like viruses, and both influenza B viral lineages (B/Florida/4/2006-like [B/Yamagata-lineage] and B/Malaysia/2506/2004-like [B/Victoria-lineage] viruses).

**Table:** Virologic data from laboratory-confirmed World Youth Day 2008 influenza cases*

Virus type/subtype	Pilgrim country of origin, no. infections					Total no. infections
	Australia	Europe	Oceania	North America	Other region or unknown	
Influenza A (H1N1) (A/Brisbane/59/2007–like) oseltamivir resistant	14	6 (Germany, Czech Republic, France, Italy, Poland)	0	0	4	24
Influenza A (H1N1) (serotype unknown) oseltamivir sensitive	1	9 (Germany, Italy)	0	0	3	13
Influenza A (H3N2) (A/Brisbane/10/2007–like)	2	9 (Germany, Italy, Slovakia, Spain)	0	2 (USA)	1	14
Influenza A (subtype/serotype not available)	2	4 (Germany, France)	0	2 (USA)	2	10
Influenza B (B/Malaysia/2506/2004–like)	2	0	7 (Solomon Islands, New Zealand, Tonga, PNG)	3 (USA, Canada)	1	13
Influenza B (B/Florida/4/2006–like)	2	0	0	1 (USA)	1	4
Influenza B (serotype not available)	6	0	7 (Solomon Islands, PNG)	3 (USA, Canada)	6	22
Total	29	28	14	11	18	100

*PNG, Papua New Guinea.

**Figure 1:**
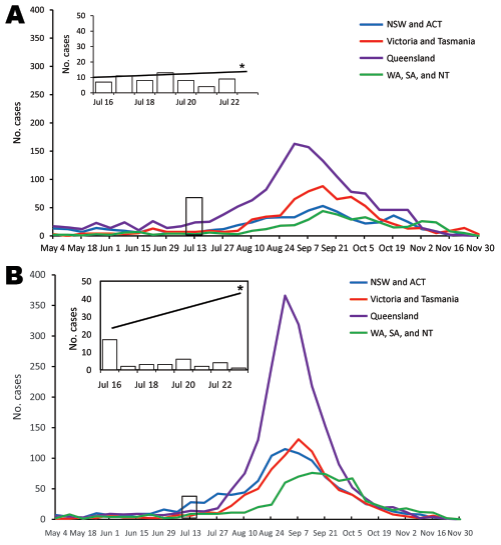
Laboratory-confirmed influenza A (A) and B (B) cases during World Youth Day 2008 (WYD2008; insets) compared with national seasonal influenza data (main graphs). Data are presented as the number of laboratory-confirmed cases per day for WYD2008 and per week for national influenza surveillance. Because laboratory methods to detect community influenza activity vary between different states, the relative effects of influenza in each state are not comparable. NSW, New South Wales; ACT, Australian Capital Territory; WA, Western Australia; SA, South Australia; NT, Northern Territory. *Background rate of laboratory-confirmed influenza for NSW/ACT included for comparison. †National data are inclusive of influenza cases diagnosed by antigen detection, nucleic acid testing, and viral isolation.

**Figure 2:**
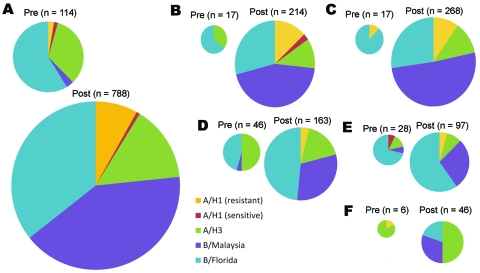
Relative effects of different influenza viruses before (pre) and after (post) World Youth Day 2008 for A) Australia; B) Western Australia; C) South Australia and Northern Territory; D) Queensland; E) New South Wales and Australian Capital Territory; and F) Victoria and Tasmania. The size of each pie chart is approximately proportional to the number of virus isolates analyzed from each region.

This outbreak occurred in the context of low seasonal influenza activity in Australia (Figure 1). An increase in influenza A and B activity was identified in all Australian states and territories in the weeks after WYD2008. Influenza isolates from WYD2008 were compared with a representative sample of national seasonal influenza isolates (12.7% of the total Australian 2008 laboratory-confirmed influenza cases). Before WYD2008, influenza (H3N2) A/Brisbane/10/2007-like and B/Florida/4/2006-like viruses were the predominant early season viral subtypes/strains observed Australia-wide (33.3% and 58.8%, respectively; Figure 2). Subsequent to WYD2008, B/Malaysia/2506/2004-like and B/Florida/4/2006-like viruses were the most frequently identified influenza strains (41.0% and 35.7%, respectively).

**Figure 3:**
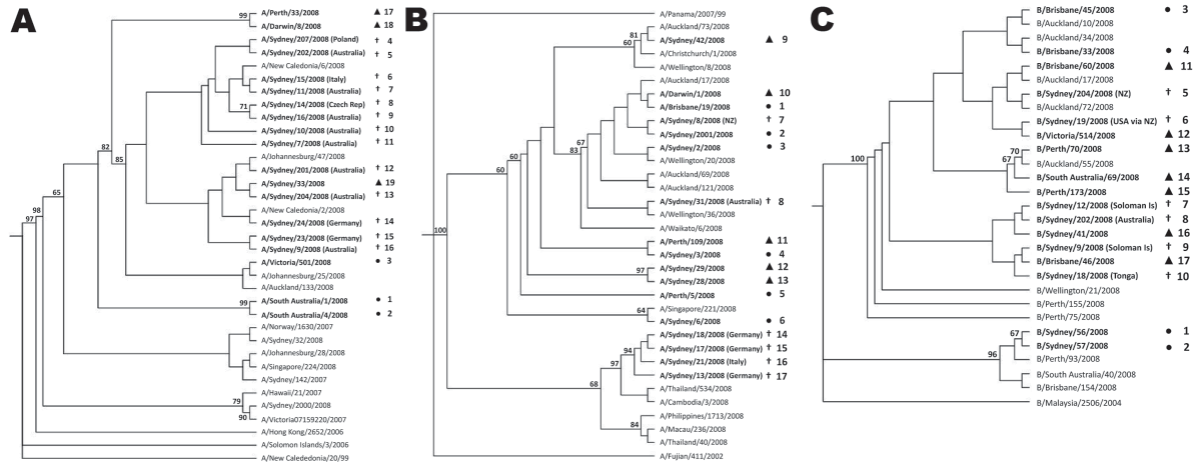
Phylogenetic trees illustrating relatedness of hemagglutinin sequences from influenza A (H1N1) (A), A (H3N2) (B), and B/Malaysia-like viruses (C) from pre–World Youth Day 2008 (WYD2008) Australian isolates (●), WYD2008 isolates (†), post-WYD2008 Australian isolates (▲), and related international isolates. Trees were constructed by using maximum-likelihood (DNAml) in PHYLIP. Only bootstrap values >60 are included.

The genetic relatedness of WYD2008 viruses to pre- and post-WYD2008 viruses was examined by sequence alignment of the HA gene (Figure 3) and NA gene (data not shown).

Before WYD2008, oseltamivir-resistant influenza A (H1N1) Brisbane/59/2007-like viruses were uncommon in Australia, and only 3 cases had been identified (Figure 2). Two cases were observed in South Australia 5 weeks and 3 days before WYD2008 (Figure 3, panel A, isolates 1–2). The remaining case was identified in Victoria in a returned traveler 5 days before WYD2008 (Figure 3, panel A, isolate 3). No obvious epidemiologic links were identified between pre-WYD2008 and WYD2008 oseltamivir-resistant influenza A (H1N1) viruses. Furthermore, isolates 1–3 appeared distinct from WYD2008 isolates. All WYD2008 influenza A (H1N1) isolates clustered relatively closely (Figure 3, panel A, isolates 4–16) and shared sequence homology with other 2008 Southern Hemisphere oseltamivir-resistant isolates. Epidemiologic data showed that most Australian and European pilgrims infected with oseltamivir-resistant A (H1N1) had traveled from Victoria to Sydney in the week before WYD2008. Despite this, oseltamivir-resistant A (H1N1) viruses were responsible for <10% of typed influenza virus infections. (Figure 2; Figure 3, panel A, isolates 17–19).

Genetic analysis of WYD2008 influenza A (H3N2) A/Brisbane/10/2007–like viruses demonstrated 2 distinct phylogenetic groups. The first influenza A (H3N2) cluster was obtained from Australian and New Zealand pilgrims only and was genetically related to sporadic Australian pre- and post-WYD2008 influenza isolates (Figure 3, panel B: pre-WYD2008, isolates 1–6; WYD2008 Australian/NZ cluster, isolates 7–8; post-WYD2008, isolates 9–13). The second phylogenetic group included influenza A (H3N2) viruses isolated from pilgrims from Germany and Italy (Figure 3, panel B: isolates 14–17; WYD2008 European cluster) and appeared to be closely related to influenza strains found in Southeast Asia in 2008. No evidence of similar strains was found in Australia before or after WYD2008.

Before WYD2008, only 4 cases of influenza B/Malaysia–like virus infection were identified Australia-wide (Figure 2). Two isolates from NSW were found to be distinct from WYD2008 isolates by sequence analysis (Figure 3, panel C, isolates 1–2). The remaining viruses (Figure 3, panel C, isolates 3–4) were identified in Queensland 2 and 5 days before WYD2008 and appeared to be closely related to WYD2008 isolates (Figure 3, panel C, isolates 5–10). No epidemiologic links to WYD2008 cases were identified. Pilgrims from the Solomon Islands sought treatment for a febrile illness at rural medical facilities 5 days before WYD2008, while they were staying close to the NSW/Queensland border. Subsequent review suggests that these illnesses were most likely influenza. WYD2008 pilgrims with confirmed influenza B/Malaysia–like virus infection were identified among contacts of these original clinical case-patients. Sequence analysis of WYD2008 isolates (Figure 3, panel C, isolates 5–10) clustered closely with isolates obtained throughout Australia post-WYD2008 (Figure 3, panel C: isolates 11–17). Because several pilgrims infected with influenza B/Malaysia-like viruses either lived or traveled through New Zealand en route to Sydney, we explored the idea that the influenza B/Malaysia from New Zealand was introduced. Peak influenza B/Malaysia activity in New Zealand occurred 4 weeks before peak activity was detected in Australia (*20*). Furthermore, WYD2008 isolates clustered closely with pre- and post-WYD2008 New Zealand isolates (Figure 3, panel C). Influenza B/Malaysia-like viruses also could have been introduced into both New Zealand and Australia from Pacific Island countries.No clear trends were identified with oseltamivir-sensitive influenza A (H1N1) and influenza B/Florida/4/2006-like viruses when WYD2008 and community isolates were compared (data not shown).

## Discussion

Thousands of mass gathering events are held each year, including major sporting events, festivals, demonstrations, and pilgrimages. Mass gatherings of a scale seen with WYD2008 have the potential to create health risks for those attending and the community at large. Although outbreaks of communicable diseases during mass gatherings have been described, insufficient epidemiologic and pathogen-related data have been described to characterize the outbreak, measure the impact on the wider community, or guide management of future mass gatherings (*8*–*10*).WYD2008 presented a unique opportunity to study the effects of influenza on mass gatherings by combining epidemiologic data acquired through prospective data collection, laboratory data obtained after respiratory tract sampling, and surveillance data obtained through established Australia-wide influenza laboratory networks.

A notable influenza outbreak occurred during WYD2008, likely exacerbated by crowded living conditions, the presence of multiple circulating influenza viruses, and low immunization rates. The true effects of influenza infection are unknown because not all infected pilgrims visited clinics and respiratory tract sampling was not performed on all clinic patients. The epidemiologic and virologic data gathered during WYD2008 highlight the complexity of an influenza outbreak within a large mass gathering. At least 6 distinct viruses circulated among pilgrims during WYD2008; 2 distinct influenza A (H1N1) viruses (oseltamivir-resistant and -sensitive), 2 distinct influenza A (H3N2) viruses (Australian/New Zealand and European clusters), and 2 distinct influenza B viruses (B/Malaysia–like and B/Florida–like). Different viruses were more likely to circulate in different groups within the pilgrim population, which suggests that exposure was not random but influenced by country of origin and travel before WYD2008.

Mass gatherings with attendees traveling from overseas allow for the introduction of novel influenza viruses as well as the amplification of preexisting community strains. Among the strains identified during WYD2008, influenza A (H3N2) viruses (Australian/New Zealand cluster) and B/Florida-like viruses appear closely related to viruses that were circulating in the community before WYD2008. Although rarely isolated before WYD2008, oseltamivir-resistant influenza A/H1N1 and B/Malaysia–like viruses isolated from pilgrims are potentially related to viruses cultured from nonpilgrims in South Australia, Victoria, and Queensland, raising the possibility of local acquisition rather than introduction from overseas. Other viruses, such as influenza A (H3N2) (European cluster), were not detected in Australia before WYD2008 and thus appear to have been introduced with the pilgrims.

Exploring the influence of a mass gathering on community influenza activity is complex, especially when numerous viruses circulate during such an event. In 2008, the total number of laboratory-confirmed influenza cases in Australia was 1.9× the 5-year average yet not significantly different from the number of cases diagnosed in 2007 (*19*). WYD2008 coincided with the start of the normal influenza season in Australia, which is usually greatest between July and September (*21*,*22*). Thus, the rapid rise in influenza A and B activity in all states after WYD2008 may have occurred despite WYD2008. To add to the complexity are the differing and unpredictable consequences of each individual viral subtype/strain. This complexity is illustrated by oseltamivir-resistant A (H1N1) and the B/Malaysia-like influenza viruses, which were rarely detected before WYD2008 but responsible for 24%–34% and 13%–35% of infections in pilgrims tested. Pilgrim groups had substantial contact with nonpilgrims through travel in Australia before and after WYD2008. Despite this contact, post-WYD2008 dissemination of influenza A (H1N1) was modest compared with dissemination of influenza B/Malaysia-like viruses (A [H1N1], 8.1% of all post-WYD2008 isolates; B/Malaysia, 41.0% of all post-WYD2008 isolates).

A potential explanation for the substantial effect of influenza B/Malaysia-like viruses observed post-WYD2008 is reduced community immunity to influenza B. In 2007, influenza B was an infrequent pathogen (6.9% of total isolates typed); influenza A (H3N2) and A (H1N1) viruses were most frequently detected (58.7% and 34.4% of total isolates typed or subtyped) (*21*). In the 2003–2006 influenza seasons, influenza B was responsible for a low prevalence of disease compared with influenza A (6%–35% of total influenza isolates typed) (*22*–*25*). Influenza A (H1N1) A/Solomon Island/3/2006–like, A (H3N2) A/Brisbane/10/2007–like, and B/Florida/4/2006–like viruses were chosen to be included in the Southern Hemisphere winter 2008 influenza vaccine. Both the low rate of influenza B activity in preceding seasons and mismatch of B/Malaysia–like viruses with the vaccine strain may have contributed to the greater B/Malaysia–like virus activity seen in community influenza in 2008.

Those who plan future mass gatherings need to consider the potential for influenza outbreaks. As observed, multiple viruses may circulate among those attending, thus increasing the opportunity for the emergence of novel reassortment viruses. Public health services need to be prepared to establish clinics rapidly and likely at many locations. Rapid diagnostic testing needs to be available, and laboratories need to be prepared for a rapid influx of specimens. Because viruses may be introduced, reliance on local rates of antiviral resistance may be misleading and resistance data from other countries may not be available. The circulation of oseltamivir-resistant seasonal influenza A (H1N1) and emergence of oseltamivir-sensitive influenza A pandemic (H1N1) 2009 virus (*26*) highlight the need for rapid typing of influenza viruses during outbreaks to guide the public health response. In influenza outbreaks in which circulating viruses include resistant seasonal influenza A (H1N1) virus, reliance on oseltamivir alone is likely to be insufficient. Influenza vaccination of all those attending should be recommended before mass gatherings, especially when held during the host countries’ influenza season and given the likely emergence of antiviral resistance. Although pilgrims were encouraged to be vaccinated before attending WYD2008, the rate of recent vaccination was low. Because numerous viruses may circulate, vaccination may be insufficient to protect against all influenza strains.

This study has several limitations. First, clinics were established rapidly and involved numerous clinicians; thus, the prospectively collected data were not standardized. Second, only pilgrims who visited clinics with symptoms were assessed, and the proportion of the pilgrim population that had an influenza-like illness or asymptomatic infection is unknown. Third, the actual proportions of viruses detected and the pilgrim populations infected may differ from those described because respiratory tract sampling was restricted at several clinics after the identification of an outbreak. Fourth, Australian influenza type/subtype data used for comparison may be influenced by a referral bias from state-based reference laboratories. Finally, only representative isolates sent to the WHO Collaborating Centre underwent sequence analysis, so further related or unrelated isolates may not have been analyzed.

After epidemiologic and virologic assessment of the WYD2008 outbreak, we highlight the complexity of influenza outbreaks that occur during mass gatherings with numerous viruses co-circulating among attendees. Mass gatherings enable introduction of novel influenza strains into the local population and the amplification of circulating local seasonal influenza strains, or both, thereby increasing the opportunity for novel reassortment influenza viruses to emerge. This introduction or amplification of viruses in contained outbreaks may alter seasonal influenza activity subsequent to a mass gathering. The resultant effect on seasonal influenza activity is influenced by many competing forces, including population movement and preexisting immunity, and thus remains unpredictable. The potential for a substantial influenza outbreak needs to be considered before all mass gathering events, particularly when hosted during the months of peak influenza activity (*27*,*28*). Greater flexibility by public health and hospitals is required to appropriately manage and contain these outbreaks.
